# Enhancing Corrosion and Wear Resistance of AA6061 by Friction Stir Processing with Fe_78_Si_9_B_13_ Glass Particles

**DOI:** 10.3390/ma8085084

**Published:** 2015-08-07

**Authors:** Lingyu Guo, Yan Liu, Kechang Shen, Chaoqun Song, Min Yang, Kibuem Kim, Weimin Wang

**Affiliations:** 1 Key Laboratory for Liquid-Solid Structural Evolution and Processing of Materials, Ministry of Education, Shandong University, Jinan 250061, China; E-Mails: guolingyusdu@gmail.com (L.G.); ly7623@sdjzu.edu.cn (Y.L.); kechangshsdu@gmail.com (K.S.); songchaoqun123@gmail.com (C.S.); miny@sdu.edu.cn (M.Y.); 2 Hybrid Materials Center (HMC), Faculty of Nanotechnology and Advanced Materials Engineering, Sejong University, 209 Neungdong-ro, Gwangjin-gu, Seoul 143-747, Korea; E-Mail: kbkim@sejong.ac.kr

**Keywords:** aluminum alloy, Fe_78_Si_9_B_13_, friction stir processing, corrosion, wear

## Abstract

The AA6061-T6 aluminum alloy samples including annealed Fe_78_Si_9_B_13_ particles were prepared by friction stir processing (FSP) and investigated by various techniques. The Fe_78_Si_9_B_13_-reinforced particles are uniformly dispersed in the aluminum alloy matrix. The XRD results indicated that the lattice parameter of α-Al increases and the preferred orientation factors *F* of (200) plane of α-Al reduces after friction stir processing. The coefficient of thermal expansion (CTE) for FSP samples increases at first with the temperature but then decreases as the temperature further increased, which can be explained by the dissolving of Mg and Si from β phase and Fe_78_Si_9_B_13_ particles. The corrosion and wear resistance of FSP samples have been improved compared with that of base metal, which can be attributed to the reduction of grain size and the CTE mismatch between the base metal and reinforced particles by FSP, and the lubrication effect of Fe_78_Si_9_B_13_ particles also plays a role in improving wear resistance. In particular, the FSP sample with reinforced particles in amorphous state exhibited superior corrosion and wear resistance due to the unique metastable structure.

## 1. Introduction

Aluminum and its alloys are used extensively in numerous fields due to their low densities and high strength to weight ratio [1,2]. Nevertheless, their poor resistance to wear and corrosion causes certain limitations for their application [3]. Metal matrix composites are a new type of material that exhibit good wear and corrosion resistance properties as compared to the matrix [4,5].

As wear and corrosion are surface-dependent degradation, they can be improved by a suitable modification of surface microstructure and/or composition. A proper technique can be employed to refine the microstructure and homogeneous dispersion of reinforcements on metallic surface. With conventional surface modification techniques it is difficult to improve the dispersion of reinforcement particles on the metal surface [6]. According to previous research [7,8], thermal spraying and laser beam techniques were used to prepare surface composites, but the creation of an unfavorable phase contributed to the degeneration of the properties. These techniques were operated at high temperature and it was difficult to avoid a reaction between the reinforcements and the matrix, which leads to the formation of an adverse phase. Thus, a fabrication process below the melting temperature of the matrix is critical to avoid the complications mentioned above.

Recently, friction stir processing (FSP), a successful surfaced modification technique derived based on the principles of friction stir welding (FSW) by Mishra *et al.* [9], has attracted much attention [10,11]. During the processing, a non-consumable rotating tool is plunged into the interface between two plates to be joined and traversed along the line of the joint with a specially designed pin and shoulder. The severe plastic deformation and material flow in the stirred zone can be utilized to achieve bulk alloy modification via mixing of other elements or the second phase into the stirred alloys, accompanied with significant grain refinement [9]. Friction stir processing is a solid state processing technique to obtain surface composites. In previous studies [12,13,14,15], the most widely used reinforcements are ceramics, such as Al_2_O_3_ or SiC in the conventional metal matrix composites. There is little research about using amorphous alloy as a reinforced material. The amorphous materials have superior properties such as high strength, high hardness, and excellent corrosion resistance due to their disordered and metastable structure [16,17,18,19].

In the present study, we focus on the development of a new AA6061-T6 alloy matrix reinforced with Fe_78_Si_9_B_13_ particles, which are fabricated by a friction stir process. The microstructure, corrosion resistance, wear behavior, and magnetic properties of the composites and base metal are investigated by different techniques.

## 2. Experimental Procedure

In this study, the base metal employed is a 4 mm thick aluminum alloy AA6061-T6, which has a chemical composition of (in wt.%) Mg: 0.95; Si: 0.54; Fe: 0.22; Mn: 0.13; Cu: 0.17; Cr: 0.09; Zn: 0.08; Ni: 0.02; Ti: 0.01; Al: balance [20]. Using manual drilling machines we created a series of blind holes in the aluminum alloy surface, with a diameter and depth of 3 mm. The reinforced particles are Fe_78_Si_9_B_13_ metal glasses after annealing and high energy milling processes. The annealing temperatures are 300 °C and 700 °C, respectively. The milling parameters are selected as following: the ratio of ball to powder is 10:1, frequency is 20 Hz, and the milling temperature is −196 °C. The FSP machine was used for fabricating *in situ* hybrid surface composites. The parameters for FSP include a traveling speed (v) of 45 mm/min, a rotational rate (w) of 900 r/min, a descending distance of 3 mm, and four stirrings. The base metal and the composites reinforced with particles annealed at 300 °C and 700 °C were named S000, S300, and S700, respectively.

The phase components of samples were investigated by X-ray diffractometer with Cu target (D/MAX 2500/PC, Rigaku, Tokyo, Japan, Cu Kα, *λ* = 0.154056 nm) and Co target (X'Pert PRO, PANalytical, Almelo, the Netherlands, Co Kα, *λ* = 0.178897 nm). The lattice constant is calculated by the extended Bragg equation [21]:

(1) $$a_{0}=\frac{\lambda }{2sin\theta }\sqrt{h^{2}+k^{2}+l^{2}},$$

where *λ* is the radiation wavelength; *θ* is the diffraction angle; and *h*, *k*,and *l* are the crystal plane indices. In present samples, the preferred orientation factors *F* of (200) plane of α-Al are investigated by the Lotgering method [22]:

$$F=\frac{P-P_{0}}{\text{1}-P_{0}},\;P=\frac{\sum I_{\text{(}h\text{00)}}}{\sum I_{\text{(}hkl\text{)}}}\;\text{and}\;P_{0}=\frac{\sum I_{0\text{(}h\text{00)}}}{\sum I_{0\text{(}hkl\text{)}}},$$

where *I*_(*h*00)_ and *I*_(*hkl*)_ are the integral intensities of diffraction peaks for composites samples and randomly oriented samples, respectively; and *P* and *P*_0_ are the ratios of integral intensities of (*h*00) planes to those of all (*hkl*) planes for composites samples and randomly oriented samples, respectively.

The microstructure of samples was examined by scanning electron microscopy (SEM, SUPRA55, Zeiss, Germany). Thermal expansion curves were measured by a dilatometer (DIL, Netzsch 402C, Netzsch, Shanghai, China) with a constant heating rate 20 K/min, performed under flowing high purity argon to protect the samples from oxidation.

The electrochemical measurements were carried out by CHI660E advanced electrochemical workstation with a scanning rate of 1 mV/s at room temperature. There was a typical three-electrode system in electrochemical measurements: working electrode, platinum counter electrode and Hg|Hg_2_Cl_2_ (SCE) reference electrode. The electrolyte is 3.5 wt% NaCl solutions.

The wear behavior of the base metal and hybrid surface composites was measured using a wear apparatus (M-2000, Yinuo Testing Machine, Jinan, China) at room temperature. The wear test was conducted at a rotation rate of abrasive wheel of 200 r/min, normal force of 120 N, and sliding time of 10 min.

## 3. Results

**Figure 1:**
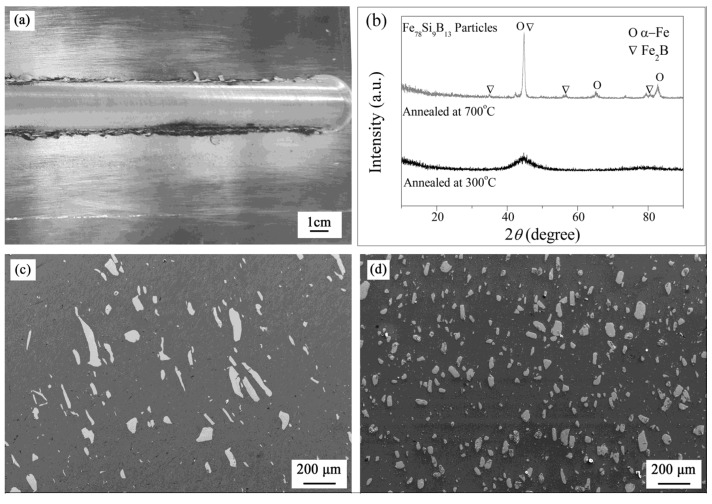
Topography of FSP samples: (**a**) Macrograph of samples after friction stir processing; (**b**) XRD patterns of the reinforced particles; (**c**) SEM photomicrograph of S300; (**d**) SEM photomicrograph of S700.

Figure 1a illustrates an overview of the side of the FSP plate in contact with the shoulder tool. This surface shows semicircular features, similar to those induced by a conventional milling process without cracking and other disfigure. The XRD patterns of the reinforced particles are also shown in Figure 1b, explaining that the particles annealed at 300 °C are in the amorphous state, and those annealed at 700 °C are in the crystalline state. Figure 1c,d show the morphology of the samples with the reinforced particles annealing at 300 °C and 700 °C, denoted as S300 and S700, respectively. It can be found that the reinforced particle is relatively uniform dispersed in the aluminum alloy matrix, and the size of the particle annealed at 700 °C is smaller than that of the particle annealed at 300 °C.

**Figure 2:**
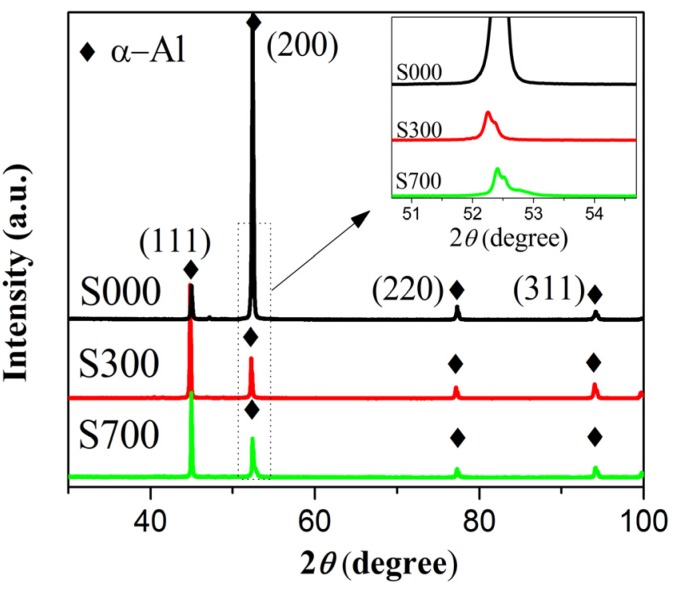
The XRD diffraction patterns for base metal (S000) and FSP samples (S300 and S700).

Figure 2 shows the XRD patterns of AA6061 FSP samples with Fe_78_Si_9_B_13_ particles (S300 and S700) as well as the base alloy (denoted S000). The main diffraction peaks are α-Al phases, and the other intermetallic phases are not found due to the less content. The (111), (200), (220), and (311) peaks of α-Al are easily identified, and the intensity of the (200) peak changed obviously. The inset shows that the peak of diffraction (2*θ*) of (200) plane of α-Al in S300 shifts to the left after FSP. For clarity, the angle of diffraction (2*θ*), lattice parameter *a*_0_, and preferred orientation factors *F* of (200) plane of α-Al are listed in Table 1. According to Equation (1), the lattice constants of the (200) plane of α-Al for the three samples are in the order S300 > S700 > S000. However, the preferred orientation factors *F* of the (200) plane of α-Al for the samples are in the order S300 < S700 < S000, implying that the initial rolling texture was destroyed during friction stir processing. In addition, the variation of XRD data with the Co target is confirmed by that with the Cu target.

**Table 1:** The preferred orientation factors *F*, lattice parameter *a*_0_, and angle of diffraction (2*θ*) of the (200) plane of α-Al of samples obtained from X-ray diffraction.

Sample	2*θ* (degree)	*a*_0_ (angstrom)	(*a*_0_ *− a*_0_*)/*a*_0_* (%)	*F*	*F’*
S000	52.434	4.0495	0.003	0.7530	0.7462
S300	52.252	4.0626	0.330	0.0031	0.0293
S700	52.406	4.0515	0.052	0.1023	0.1009

Notes: 2*θ*, *a*_0_, and *F* were tested by the Co target; *F*’ was tested by the Cu target; and *a*_0_* was the lattice constant of pure Al.

**Figure 3:**
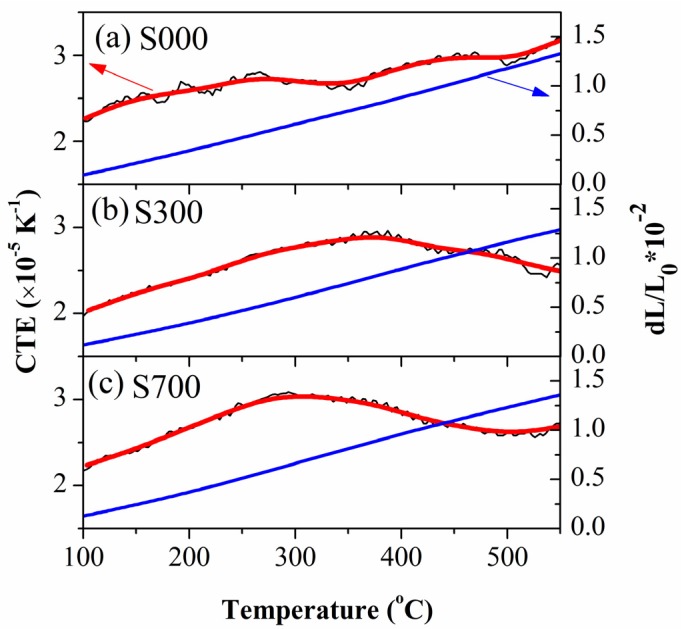
Thermal expansion curves (blue) and the variation of experimental coefficient of thermal expansions (CTE) (red) for (**a**) base metal (S000) as well as (**b**) S300 and (**c**) S700 FSP samples.

The thermal expansion curves and the variation of experimental coefficient of thermal expansions (CTE) obtained for base metal and FSP samples are shown in Figure 3. The three samples have an approximately linearly varying trend: the thermal expansion ratio increases with increasing temperature. In addition, the CTE of S000 increases with increasing temperature, but there is a plateau at about 300 °C. By contrast, the CTE of FSP samples at first increases with the temperature but then decreases as the temperature is further increased, and the temperature at which the CTE begins to decrease for S300 is about 350 °C, which is higher than that for S700, which is about 300 °C. Furthermore, the maximal CTEs for S300 and S700 are 2.87 × 10^−5^ K^−1^ and 3.04 × 10^−5^ K^−1^, respectively, and that of S000 at platform is 2.71 × 10^−5^ K^−1^, which is lower than the former values. Moreover, at 550 °C, the CTE of S000 is about 3.18 × 10^−5^ K^−1^, which is higher than that of S300 and S700, which are 2.48 × 10^−5^ K^−1^ and 2.71 × 10^−5^ K^−1^, respectively, *i.e.*, in the order S300 < S700 < S000.

**Figure 4:**
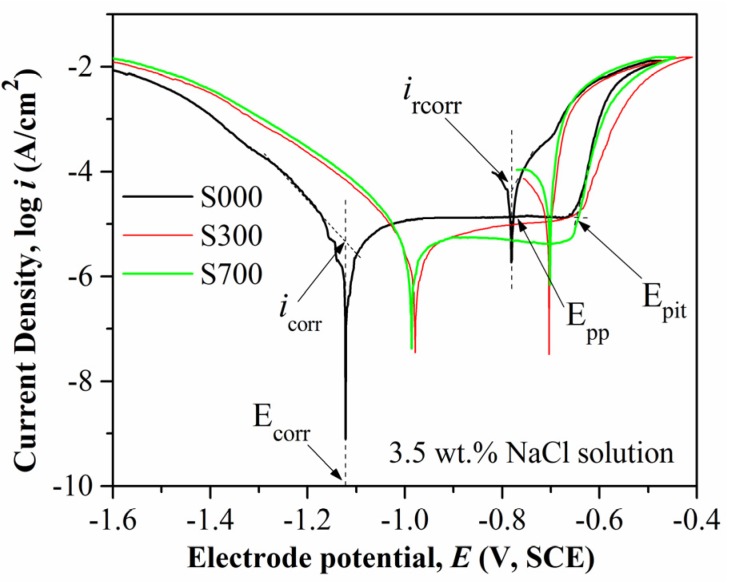
Cyclic anodic polarization curves of base metal (S000) and FSP samples (S300 and S700).

**Figure 5:**
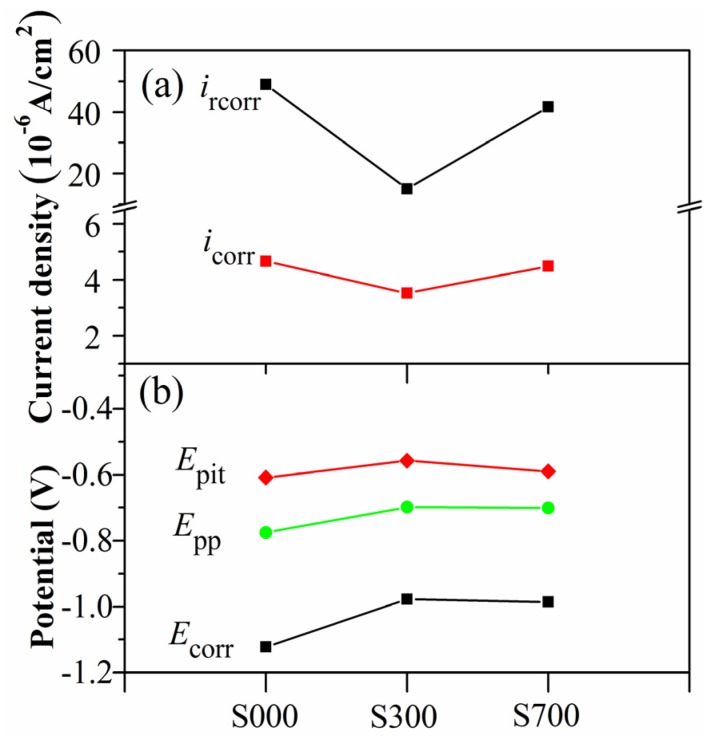
Parameters of the polarization obtained from Figure 4: (**a**) corrosion current density (*i*_corr_) and recorrosion current density *i*_rcorr_; (**b**) corrosion potential (*E*_corr_), pitting potential (*E*_pit_) and protection potential (*E*_pp_).

The cyclic anodic polarization curves of the base metal and FSP samples with a scanning rate of 1 mV/s, obtained in 3.5 wt% NaCl solutions, are shown in Figure 4. As can be seen from the curves, an obvious current plateau is observed in each curve, which is associated with passive film formation during the anodic polarization. The electrochemical parameters such as corrosion potential (*E*_corr_), corrosion current density (*i*_corr_), pitting potential (*E*_pit_), protection potential (*E*_pp_), and recorrosion current density (*i*_rcorr_) are collected in Figure 5. The *E*_corr_ of the three samples are also in the order S300 > S700 > S000, and *i*_corr_ is quite the opposite, indicating that the anodic reaction of FSP samples occurs with more difficulty in the electrochemical test. Furthermore, the *E*_pit_ and *E*_pp_ of S300 and S700 are more positive than that of S000, and S300 has the highest *E*_pit_ and *E*_pp_, which is consistent with the measured *E*_corr_. Here, the concept of protection potential (*E*_pp_) represents the potential at which pre-existing pits cease to propagate, that is, all the propagating pits are repassivated [23]. The *E*_pit_ and *E*_pp_ are related to pitting corrosion resistance, and the higher *E*_pit_ and *E*_pp_ indicate higher resistance to pitting corrosion. The *i*_rcorr_ represents the current density at which the samples incur corrosion after the repassivation region. The *i*_rcorr_ of FSP samples is lower than base metal, and the *i*_rcorr_ of S300 is the lowest, *i.e.*, in the same order of *i*_corr_. Combined with the above measured electrochemical parameters, it is found that the corrosion resistance of the three samples is in the order S300 > S700 > S000.

**Figure 6:**
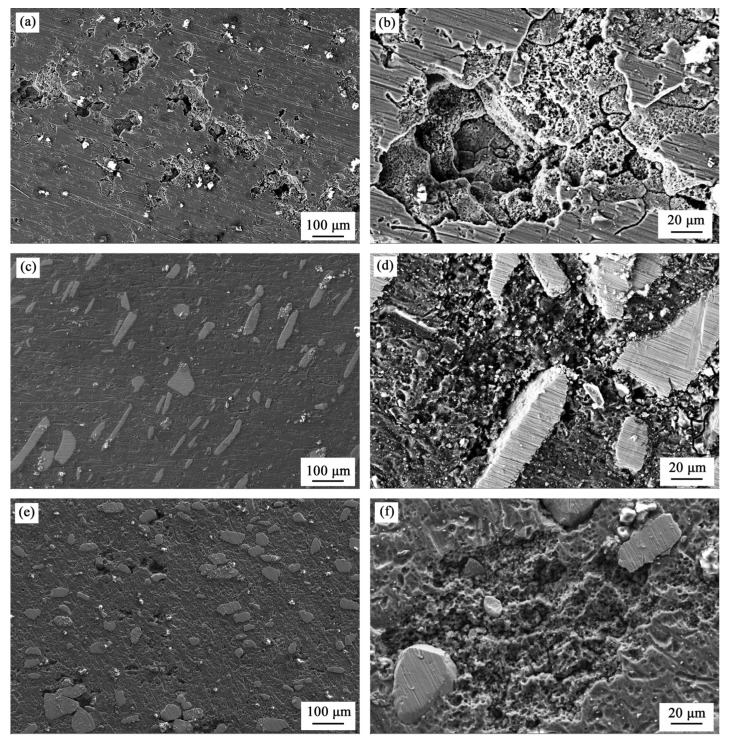
SEM micrographs of electrochemically corroded surfaces: (**a** and **b**) S000; (**c** and **d**) S300; (**e** and **f**) S700.

The SEM images of electrochemical corroded surfaces of base metal and FSP samples are shown in Figure 6. The measured samples were polarized until −0.4 V_SCE_ with a scanning rate of 1 mV/s and without backward scanning (the polarization curves are not shown here). Many corrosion pits of 50 μm in diameter appear on the surface of S000; by contrast, there are fewer corrosion pits of smaller size (~20 μm) on the surface of S300 and S700. With further investigation, the pits on the surface of S300 are shown to be much sparser than that of S700. The pits on the base metal are deeper than on the FSP samples, accompanied by obvious cracking.

**Figure 7:**
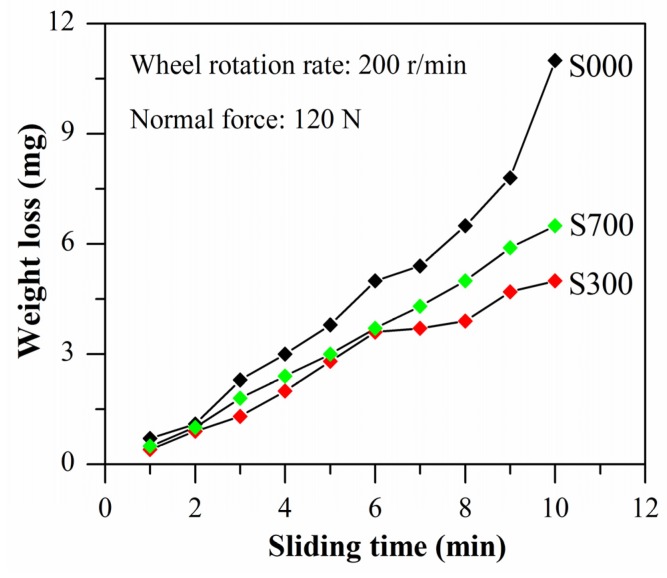
Weight loss of samples as a function of sliding time for base metal (S000) and FSP samples (S300 and S700).

**Figure 8:**
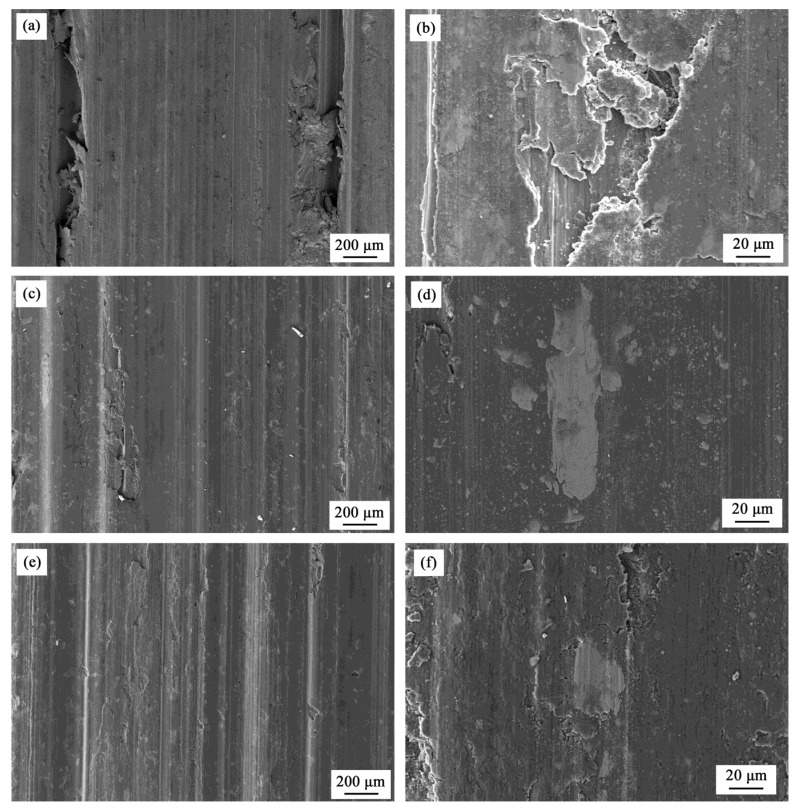
SEM micrographs of worn tracks of samples: (**a** and **b**) S000; (**c** and **d**) S300; (**e** and **f**) S700.

Figure 7 shows the weight loss of S000, S300, and S700 samples as a function of sliding time. It is observed that their weight losses increase monotonically with the sliding time, and when the sliding time reaches 7 min, the weight loss of S000 increases more quickly than that of S300 and S700. Overall, the wear resistance of three samples are ranked in the order S300 > S700 > S000, which is consistent with their corrosion resistances (Figure 4 and Figure 6). The SEM micrographs of worn tracks of the three samples are shown in Figure 8. The worn surface of the base metal (S000) shows a large amount of plastic flows. The extent of wear in S300 and S700 is lower than that in S000, *i.e.* their surfaces are smoother and the groove is shallower compared with S000. The higher magnified view of the wear pits shows that the flaking of the base metal is more serious than with the FSP samples, indicating that the worn debris formation is easier at the base metal (Figure 8b,d,f). In addition, there are a lot of fine particles in the size of several microns around the Fe_78_Si_9_B_13_ particles (Figure 8d,f) and there exist white particles that impede the worn pit extend along the sliding direction (Figure 8c), indicating that the added Fe_78_Si_9_B_13_ particles play a role in improving the samples’ wear resistance.

## 4. Discussion

### 4.1. Microstructure and Thermal Expansion Behavior of FSP Samples

It is well known that grain refinement in metals and alloys can be realized by friction stir processing (FSP), which was derived based on the principles of friction stir welding (FSW) [9,10] and is similar to the grain refining effect of severe plastic deformation (SPD) [24]. Hence, FSP can be considered one of the variants of the severe plastic deformation (SPD) technologies, which is also discussed in recent reports [25,26]. It has been reported that the SPD treatment accelerates the atomic diffusion inside the processed material, which attributes to the intensive vacancy generation during deformation [27,28,29]. Moreover, the solvus temperature of needle-shape precipitate (β”) in 6061 aluminum alloys is about 353 °C, which is lower than the maximal temperature during FSP [30], so the β” can easily dissolve into the matrix during FSP. In addition, the β” mainly contains Mg and Si atoms and the dissolution of Mg and Si into aluminum matrix could increase the lattice constant of α-Al [31], thus it is understood that the lattice parameters of S300 and S700 increase after FSP compared with S000 (Figure 2 inset and Table 1). Furthermore, it is reported that the initial rolling texture is destroyed by the deformation during the FSP [32,33,34]. It is explained that the preferred orientation factor *F* of the (200) plane in α-Al for S300 and S700 is higher than that of S000 (Figure 2 and Table 1).

In the S300 sample, the Fe_78_Si_9_B_13_ particles annealed at 300 °C are in the amorphous state, while the Fe_78_Si_9_B_13_ particles have crystallized completely during the annealing process at 700 °C (Figure 1b). In the amorphous state, the atoms are metastable and in a random order [19], therefore the Si in Fe_78_Si_9_B_13_ is apt to dissolve into the aluminum matrix and form a solid solution during FSP. Furthermore, the dissolution of Si could also induce severe lattice distortion and dislocation, which may lead to the dissolution of more Mg and Si into the aluminum matrix. In the S700 sample, the influence of the Fe_78_Si_9_B_13_ particle annealed at 700 °C on the lattice parameter is weaker due to its crystalline structure. As a consequence, the lattice constant *a*_0_ of α-Al in S300 is larger than that in S700 (Table 1). Meanwhile, compared with S700, the lower *F* of S300 indicates a severer recrystallization and then a severer destruction of rolling texture, which may result in a severer lattice distortion and dislocation in S300 and a higher dissolution degree of Mg and Si. This is also confirmed by the fact that the *a*_0_ of α-Al in S300 is higher than that in S700 (Table 1).

According to the micro-mechanical model [35], the initial compression stress of a sample induced by CTE mismatch of the constituents is relieved with increasing temperature and causes an increment in the coefficient of thermal expansion (CTE). We determined the initial increment of CTE of the three measured samples (Figure 3). Furthermore, the CTEs for the three samples increase at first, which is also related to the dissolution of the β or β” phase, which mainly contains Mg and Si due to the positive contribution of Mg to CTE and *a*_0_ of α-Al solution [31,36,37]. During the cooling process after FSP, the shrinkage degree of base metal was larger than that of the reinforced particles, because the CTE of added particles is less than that of the aluminum matrix alloy, resulting in compressive stress in both the base metal and the added particles [38]. It is understood that the initial CTE of S300 and S700 is lower, and the CTE increment in the beginning stage is higher compared with S000 (Figure 3). Moreover, the maximal CTEs for S300 and S700 are 2.87 × 10^−5^ K^−1^ and 3.04 × 10^−5^ K^−1^, respectively, and the first maximal of S000 is 2.71 × 10^−5^ K^−1^, which is lower than the former values, indicating a higher ability to dissolve Mg for S300 and S700. This is consistent with the *a*_0_ variation of measured samples (Table 1). In addition, the temperature at which the CTE begins to decrease for S300 is higher than that for S700, which is due to increased Mg dissolution into the matrix during FSP for S300, which is also reflected in the data of lattice parameters (Table 1). The decrease of CTE for FSP samples after the maximal CTE is related to the Si dissolution into the matrix, supplied by Fe_78_Si_9_B_13_ particles with a negative contribution to CTE of matrix [39]. Moreover, at 550 °C, the CTE of S300 is lower than that of S000 and S700, which is related to shrinkage of the amorphous Fe_78_Si_9_B_13_ particles in S300 crystallized during heat processing.

### 4.2. Corrosion and Wear Behavior of FSP Samples

After friction stir processing (FSP), the grain size decreased due to dynamic recrystallization [34], which occurs early on in the alloys after SPD [40,41]. The dynamic recrystallization process has undergone a series of paths [42], which involves the dissolution introduction, dynamic recovery, and continuous dynamic recrystallization. It has been reported that the decrease of grain size can reduce the corrosion rate, that is, it improves the corrosion resistance, which is attributed to an ability of fine-grained materials to passivate more readily and to physical breakdown in the second phase of the intermetallic particles that operated as efficient local cathodes and sites for initiation of localized attack [43,44]. Due to the decrease of grain size by dynamic recrystallization during FSP [33,34], the FSP will improve the corrosion resistance of the aluminum alloy, which is in accordance with Rao’s research [45]. On the other hand, in the weld head and its vicinity the residual FSP stresses are either parallel or perpendicular to the weld direction; these are compressive stresses due to the CTE mismatch between the base metal and particles [38,46]. The compressive residual stress can improve the corrosion resistance of the surface by reducing the tendency of cracking [34,47,48]. Hence, S300 and S700 have more positive *E*_pit_ and *E*_pp_ than S000 and fewer cracks than S000 (Figure 4, Figure 5 and Figure 6). Because the CTE of Fe_78_Si_9_B_13_ in the amorphous state is less than that in the crystallized state [38], the S300 endured larger compressive stress than S700. Moreover, it has been reported that the amorphous samples have a better corrosion resistance than the crystallized ones [49,50]. Hence, it appears that S300 has more positive *E*_corr_, *E*_pit_, and *E*_pp_ than S700 (Figure 4, Figure 5 and Figure 6).

The presence of added particles converting the wear mode of FSP samples from two bodies to three bodies reduces the loss of wear further by acting as a solid lubricant [5,14,51]. Apparently, the fine powders of the size of several microns around the Fe_78_Si_9_B_13_ particles possibly act as lubricants (Figure 8d,f). In addition, the residual stress after FSP is compressive stress [46], and moderate levels of compressive residual stress have beneficial effects on wear resistance by delaying crack initiation and growth [52]. Furthermore, the smaller grain was conducive to formation of a tribological transformed structure (TTS) layer, and produced shorter delamination cracks in the TTS layer than the larger one [53]—that is, the ultrafine-grained microstructures exhibit markedly enhanced wear resistance relative to the coarse-grained counterpart. Consequently, for the particles located at the end of worn pits, the wear loss of S300 and S700 is lower than S000 (Figure 7). It is known that the amorphous Fe_78_Si_9_B_13_ is metastable and has a drastic stress-induced flow at about 400 °C during the heating process [19,54], which possibly accounts for it acting as a better lubricant (Figure 7). As discussed previously, the difference of CTE for added particles leads to a larger compressive stress in S300 than in S700. Hence, it is shown that S300 has better wear resistance than S700 (Figure 7). In other words, amorphous Fe_78_Si_9_B_13_-reinforced particles can improve the wear and corrosion resistance of AA6061 samples.

## 5. Conclusions

The microstructure, thermal expansion behavior, corrosion behavior and, wear resistance of AA6061-T6 with Fe_78_Si_9_B_13_ glass particles by friction stir processing have been investigated (the base metal is denoted as S000, and the FSP samples with the reinforced particles annealed at 300 °C and 700 °C are denoted as S300 and S700, respectively), and it was found that:

(1) By friction stir processing, the Fe_78_Si_9_B_13_ particles are relatively uniform in terms of their dispersion into the Al alloy matrix. The *a*_0_ of α-Al for three samples is in the order S300 > S700 > S000, but the *F*_200_ for samples is the opposite. During the heating process, the CTEs of the three samples increase firstly and have a maximal value of 2.71 × 10^−5^ K^−1^, 2.87 × 10^−5^ K^−1^, and 3.04 × 10^−5^ K^−1^ for S000, S300, and S700, respectively. The CTE variation can be explained by the dissolving of Mg and Si from the β phase and Fe_78_Si_9_B_13_ particles, as well as the CTE mismatch compressive stress.

(2) The *E*_corr_ and *E*_pit_ of the three samples are also in the order S300 > S700 > S000, while *i*_corr_ is quite the opposite. The superior corrosion resistance for S300 can be attributed to the reduction of grain size by FSP and the unique metastable structure of amorphous Fe_78_Si_9_B_13_-reinforced particles. Moreover, the corrosion surface of S300 and S700 has little corrosion pits, but no apparent cracking compared to S000.

(3) The wear resistance of FSP samples has been improved compared with the base metal; in particular, the weight loss for the samples is in the order S300 < S700 < S000. The flaking of S000 after a wear test is more serious than that in S300 and S700. In addition, the worn surfaces of FSP samples are smoother and the grooves shallower compared with S000. The partial improvement of corrosion and wear resistance is ascribed to the compressive stress in FSP samples due to the CTE mismatch between the base metal and reinforced particles, and the lubrication effect of Fe_78_Si_9_B_13_ particles also plays a role in improving wear resistance.
